# Penile Metastasis-Induced Priapism as the First Sign of Lung Cancer: A Case Report and Review of the Literature

**DOI:** 10.1155/2024/1692706

**Published:** 2024-11-05

**Authors:** Rawad Abou Zahr, Eliott Nadalin, Sarah Thiry, Raquel Da Silva Maia, Axel Feyaerts, Bertrand Tombal

**Affiliations:** ^1^Urology Department, Cliniques universitaires Saint-Luc, Avenue Hippocrate 10 1200, Brussels, Belgium; ^2^Faculty of Medicine, Université Catholique de Louvain, Brussels, Belgium

## Abstract

**Background:** The penis is a relatively uncommon organ for metastases. Secondary lesions often originate from the bladder, prostate, or rectosigmoid cancers. Only a few cases have described penile lesions secondary to lung cancers, mostly as a later complication.

**Case Description:** We hereby report the case of an 86-year-old male patient who presented with a 3-week-long nonpainful priapism. A penile Doppler ultrasound and a chest and abdominal CT scan were performed, showing a left hilar lung mass as well as lesions in the liver, the adrenal glands, the pancreas, bone structures, and the penis. Penile metastasis is associated with a poor prognosis because of the frequent disseminated malignant lesions in other sites.

**Conclusion:** Malignant priapism should be suspected, especially in patients with no evident risk factors for priapism (hematological diseases, drugs, alcohol, neurological diseases, or metabolic disorders).

## 1. Introduction

Secondary penile lesions are relatively uncommon. The first ever reported secondary malignant penile lesion was published by Eberth in 1870 and originated from an adenocarcinoma of the rectum [1, 2]. Most secondary lesions originate from primary bladder, prostate, or rectosigmoid tumors and less frequently from distant visceral organs [3, 4]. Primary lung tumors tend to metastasize usually to regional lymph nodes, brain, bone, liver, adrenal glands, ipsilateral and contralateral lungs which makes the penis an unusual site [3, 4].

Only a few cases were reported in the literature where secondary penile lesions were associated with primary lung cancer [2]. Signs and symptoms such as penile mass or pain, urinary disorders, or priapism can be found [4].

Priapism by itself is described as an erection persisting for more than 4 h without any sexual stimulation or ending orgasm. Priapism is divided into ischemic (low flow) and nonischemic (high flow). The distinction between these two types is paramount since the management is entirely different. Clinically, ischemic priapism presents with a painful erection, rigid glans, and corpora. Due to the venous outflow obstruction, blood stagnation in the corpora causes hypoxemia, hypercapnia, and a drop in pH, leading to smooth muscle necrosis. The acidic environment stimulates the nociceptors, which explains the painful erection. This requires urgent management by blood aspiration with/without intracavernosal sympathomimetics to release smooth muscle contraction and restore the environment with well-oxygenated blood [5, 6].

Nonischemic priapism usually presents with a nonpainful, partial erection with flaccid glans. Unlike ischemic priapism, nonischemic priapism is not an emergency. It involves an arteriosinusoidal fistula, which does not affect the blood flow, thus keeping the tissues well-oxygenated. The risk of smooth muscle necrosis is relatively low; therefore, conservative treatment is recommended as a first-line option. If it fails, selective embolization of the fistula by arteriography can be performed [5, 6].

We hereby present the case of an 86-year-old male who presented with a 3-week nonpainful priapism as a first sign of his primary lung neoplasm by the CARE reporting checklist.

## 2. Case Presentation

**Figure 1:**
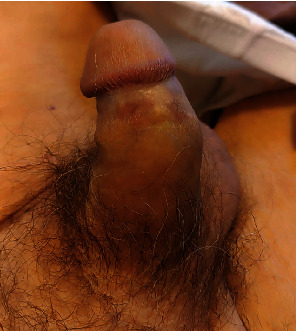
The penis in erect status with a lateral deviation.

An 86-year-old male patient presented to the emergency room for a 3-week nonpainful priapism. Our patient, a former cleaner agent, is only known for a transient ischemic incident. He has no previous surgical history. Physical examination showed a semierect nontender penis with a mild lateral deviation and a smooth glans (Figure 1). No superficial penile lesion was observed. The patient reported that he had lost normal erections almost 20 years ago. All clinical signs were in favor of a nonischemic priapism. The patient was a former smoker and reported mild upper respiratory tract symptoms as well. Lab work-ups and urine analyses were unremarkable; hence, the decision was to pursue investigations in an ambulatory setting using a penile ultrasound and a thoracoabdominal computed tomography (CT) scan looking for a malignancy.

**Figure 2:**
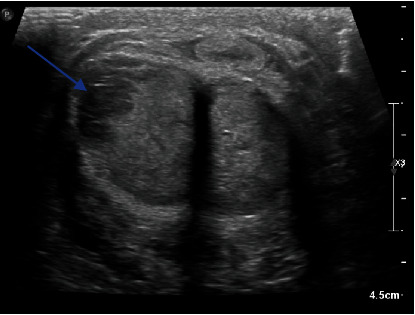
Penile ultrasound axial cuts showing tumor invasion of the corpora cavernosa on the right (blue arrow).

Doppler ultrasonography (US) of the penis was performed, revealing circumscribed hypoechogenic nodules infiltrating the albuginea of the corpus cavernosum, with a base and right-side predominance. No abnormalities were detected in the corpus spongiosum (Figure 2). A normal resistive index with a normal penile artery velocity was measured, in favor of a nonischemic situation.

**Figure 3:**
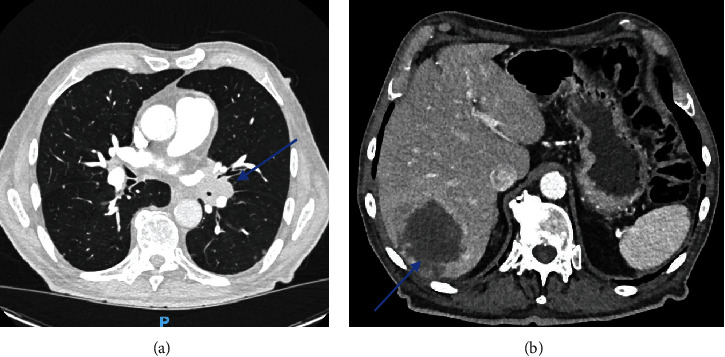
(a) Lung mass on the left hilum. (b) Liver metastasis.

Thoracoabdominal CT revealed a mass at the lung's left hilum surrounding the bronchovascular structures (Figure 3(a)). Several bilateral nodules and micronodules were observed. The largest ones were located at the right apex and right lung base. Mediastinal enlarged lymph nodes were also seen at the lower paratracheal station. The liver was also affected by several lesions; the largest was located at Segment 7, which measured 96 mm (Figure 3(b)). The left adrenal gland and pancreatic lesions were noted. Finally, two osteolytic lesions were observed on the first left rib and on the transverse process of fifth lumbar vertebra (L5). The patient was referred to the lung cancer specialist for further investigations. A pulmonary biopsy was performed, confirming adenocarcinoma of the lung. Molecular analysis of the cells showed a pGly12Cys (G12C) variant of the KRAS gene. PDL-1 was also noted as positive.

After a multidisciplinary discussion, the decision was for the patient to pursue four-cycle chemotherapy and immunotherapy by cisplatin, pemetrexed, and pembrolizumab. The patient poorly tolerated the first cycle of chemotherapy and developed febrile pancytopenia. The decision was to halt the chemotherapy and pursue the immunotherapy by pembrolizumab.

As for the penile lesions, since the patient did not have any pain, surveillance was adopted.

## 3. Discussion

Around 500 cases of penile metastases have been described in the literature since Eberth reported the first in 1870 [1, 2]. The bladder, prostate, and rectosigmoid colon were the most often primary cancers from those metastases. [3, 4]. Furthermore, lung cancer usually metastasizes to the regional lymph nodes, brain, bone, liver, and adrenal glands, as well as ipsilateral and contralateral lungs [3, 4].

According to the literature, the average age for detection of secondary penile lesions was around 61. The incidence tends to increase with age. Most reported secondary penile lesions were discovered at the time of diagnosis or developed later as a complication of lung cancer. Only few exceptions have been described, including our own, showing a penile lesion as a first sign of the primary lesion [2].

Squamous cell carcinoma and adenocarcinoma are the most commonly described histological types of lung cancer that tend to metastasize to the penis, with a significant predominance for squamous cell carcinomas. This can hypothetically be explained by a significant predominance of lung adenocarcinoma incidence in women [4, 7].

The metastatic lesions are usually located on the shaft of the penis [7]. The corpus cavernosum was also bilaterally affected in most cases. This could be related to the incomplete septum separating the corpora cavernosa, allowing communication between them. A smaller percentage of unilateral lesions at the root, foreskin, or glans have also been described [4].

Regarding pathophysiology, there are several routes of dissemination of tumor cells in the penis: the retrograde venous flow, the arterial spread, the lymphatic route, and, more rarely, direct extension of regional cancer or iatrogenic implantation during surgical procedures [4, 7]. The arterial pathway is most likely the right assumption regarding lung tumor cell dissemination [7]. This can be explained by pulmonary arteries lesions causing a systemic spread [4].

Clinically, secondary penile lesions can present with a penile mass associated with tenderness or dysuria. Urinary obstruction and hematuria are rarely observed. [4] Priapism, or malignant priapism (MP), in our case, can also be observed [4]. MP results in tumor cells' presence in the penis, whether primary (by local invasion), metastatic, or secondary to hematological cancers. MP should be considered in the differential diagnosis, especially in patients with no evident risk factors for priapism (hematological diseases, drugs, alcohol, neurological diseases, or metabolic disorders) [8].

MP can be explained either by the obstruction of the corpus cavernosum by the tumor mass preventing normal blood drainage or by secondary tumor cell thrombosis in the venous lakes, which would, in most cases, mimic low-flow ischemic priapism [4], or by secondary deposition of metastatic implants involving the corpora cavernosa and tunica albuginea mimicking priapism, as was the case in our patient.

The diagnosis is made by biopsy of the penile lesion, proving the tumor cells' presence [4, 9]. Doppler US, CT scan, or magnetic resonance imaging (MRI) of the penis are also options to show the anatomical invasion of metastasis [7, 9]. MRI is the most effective exam [7].

The choice of treatment for primary cancer responsible for the secondary penile deposits meets several criteria, including the histological type of cancer, the patient's age, general condition, size, and number of metastases [4, 7].

Penile metastasis is associated with a poor prognosis because of the frequent disseminated malignant lesions in other sites. Therefore, the treatment mostly requires a palliative therapeutic plan, usually based on chemotherapy and/or immunotherapy, depending on the primary cancer [4, 7].

It is also possible to offer local treatments for symptomatic penile lesions, such as radiotherapy, brachytherapy, or surgical resection [4].

Our patient presented a nonpainful priapism mimicking a high-flow priapism. This can hypothetically be explained by the development of an arteriosinusoidal fistula resulting from tumor extension [6, 8]. He did not present any penile mass or urine disorders. Only a pulmonary biopsy was performed, as well as a penile Doppler US proving the metastatic invasion of the penis. No penile biopsy was performed in our case as it would not have changed the treatment plan. A four-cycle chemotherapy/immunotherapy by Cisplatin, Pemetrexed and Pembrolizumab as a palliative treatment was decided at the multidisciplinary reunion of the pulmonary team. The treatment plan was then switched to monotherapy by pembrolizumab due to poor tolerance of the patient. Due to the absence of urological symptoms, no local treatment was proposed; rather, a surveillance strategy was used.

## 4. Conclusion

In conclusion, penile metastases secondary to lung cancer are rare events. They are often seen as a later complication of cancers rather than a first sign and are often signs of advanced disease [2]. They may cause penile pain, penile mass, but also MP [4]. MP can be secondary to primary penile cancer, metastasis, or hematologic cancers and should be considered as a possible cause of priapism in patients with no risk factors. MP is usually presented as low-flow priapism and requires urgent management. Therapeutic plan requires a multidisciplinary discussion but is generally palliative and symptomatic and associated with poor prognosis [8].

## Data Availability

The data used to support the findings of this report are included within the article.
